# Supplementary material for: Mechanisms of feedback inhibition and sequential firing of active sites in plant aspartate transcarbamoylase
Source: Nat Commun. 2021 Feb 11;12:947. doi: 10.1038/s41467-021-21165-9 (PMC7878868; doi:10.1038/s41467-021-21165-9)
Supplement: Supplementary file 1 — Supplementary Information [file 41467_2021_21165_MOESM1_ESM.pdf]

Mechanisms of feedback inhibition and sequential firing of active sites in plant aspartate transcarbamoylase

Leo Bellin, Francisco del Caño-Ochoa, Adrián Velázquez-Campoy, Torsten Möhlmann, Santiago Ramón-Maiques

SUPPLEMENTARY INFORMATION

Supplementary Table 1. Data collection and refinement statistics.

	**+ UMP**	**APO**	**+ PALA**	**+ PALA + CP**	**+ CP**	**F161A + UMP**	**F161A + PALA**
**Data collection**							
Space group	$P6_3$	$P2_12_12_1$	$P2_12_12_1$	$P2_12_12_1$	$P2_12_12_1$	$P6_3$	$P2_12_12_1$
Cell dimensions							
a, *b*, *c* (Å)	104.5 104.5 127.8	103.7 109.6 208.6	86.7 94.9 132.1	86.5 94.6 131.8	103.3 109.5 212.0	104.3 104.3 127.9	80.3 98.3 138.8
α, β, γ (º)	90, 90, 120	90, 90, 90	90, 90, 90	90, 90, 90	90, 90, 90	90, 90, 120	90, 90, 90
Resolution (Å)	48.38-1.71 (1.74-1.71)*	92.85-3.07 (3.18-3.07)	66.04-1.55 (1.60-1.55)	94.57-1.44 (1.46-1.44)	76.16-1.87 (1.90-1.87)	45.18-2.40 (2.49-2.40)	46.32-1.68 (1.71-1.68)
R_{merge}	0.098 (1.228)	0.14 (0.83)	0.058 (0.619)	0.085 (0.828)	0.049 (0.986)	0.194 (1.255)	0.053 (0.840)
$I / \sigma I$	14.0 (2.0)	11.2 (2.3)	15.1 (2.4)	11.1 (2.0)	18.7 (2.0)	8.5 (2.0)	18.4 (2.0)
Completeness (%)	100 (100)	99.29 (99.57)	97.4 (96.7)	100 (100)	100 (100)	100 (100)	100 (100)
Redundancy	10.3 (10.1)	5.2 (5.2)	4.8 (5.1)	6.6 (6.2)	6.7 (6.9)	10.0 (10.5)	6.6 (6.5)
**Refinement**							
Resolution (Å)	1.71	3.07	1.55	1.44	1.87	2.4	1.68
No. reflections	85,304	45,063	155,436	195,033	198,106	29,351	125,363
R_{work} / R_{free}	0.14/0.17	0.19/0.25	0.12/0.15	0.14/0.17	0.17/0.21	0.14/0.19	0.15/0.17
No. atoms (no H's)	5,608	14,521	8,584	8,261	16,723	4,948	8,439

Protein	4,985	14,466	7,658	7,492	15,187	4,894	7,617
Ligands	66	12	79	84	202	46	54
Water	557	43	847	685	1,334	8	768
B-factors	32.40	56.69	25.08	23.91	40.82	46.18	27.89
Protein	31.39	56.74	23.46	22.89	40.10	46.32	27.05
Ligand	30.83	53.33	37.38	31.79	52.75	33.78	21.51
Water	41.61	39.65	38.54	34.18	47.20	42.67	36.68
R.m.s. deviations							
Bond lengths (Å)	0.012	0.006	0.008	0.013	0.011	0.011	0.005
Angles (º)	1.59	0.72	0.99	1.54	1.693	1.782	0.857

Each dataset was collected from a single crystal.

*Values in parenthesis are for highest-resolution shell

Supplementary Table 2. Determination of ligand binding affinities by isothermal titration calorimetry (ITC)

			K_D (μM)			ΔG (kcal/mol)			ΔH (kcal/mol)			$-T\Delta S$ (kcal/mol)			n[a]
	Ligand	N[b]	Site 1	Site 2	Site 3	Site 1	Site 2	Site 3	Site 1	Site 2	Site 3	Site 1	Site 2	Site 3	
WT	PALA	1	0.59 ± 0.07	–	–	-8.5 ± 0.1	–	–	13.1 ± 0.3	–	–	-21.6 ± 0.3	–	–	1.2 ± 0.1
	UMP	3	0.21 ± 0.03	2.3 ± 0.4	1.6 ± 0.3	-9.1 ± 0.1	-7.7 ± 0.1	-7.9 ± 0.1	-2.8 ± 0.4	1.2 ± 0.4	-0.5 ± 0.4	-6.3 ± 0.4	-8.9 ± 0.4	-7.4 ± 0.4	1.1 ± 0.1
	CP	3	80 ± 20	80 ± 20	80 ± 20	-5.6 ± 0.1	-5.6 ± 0.1	-5.6 ± 0.1	-3.0 ± 0.5	-3.0 ± 0.5	3.0 ± 0.5	-2.6 ± 0.5	-2.6 ± 0.5	-2.6 ± 0.5	(1)
WT:PALA[c]	UMP	2	*with PALA*	1.2 ± 0.4	1.2 ± 0.4	*with PALA*	-8.1 ± 0.2	-8.1 ± 0.2	*with PALA*	0.4 ± 0.4	0.4 ± 0.4	*with PALA*	-8.5 ± 0.4	-8.5 ± 0.4	0.9 ± 0.1
	CP	2	*with PALA*	140 ± 50	140 ± 50	*with PALA*	-5.3 ± 0.2	-5.3 ± 0.2	*with PALA*	-5.7 ± 0.4	-5.7 ± 0.4	*with PALA*	0.4 ± 0.4	0.4 ± 0.4	(1)
F161A	PALA	1	0.12 ± 0.02	–	–	-9.4 ± 0.1	–	–	11.0 ± 0.3	–	–	-20.4 ± 0.3	–	–	1.2 ± 0.1
	UMP	–	–	–	–	–	–	–	–	–	–	–	–	–	–
	CP	3	0.73 ± 0.09	0.73 ± 0.09	0.73 ± 0.09	-8.4 ± 0.1	-8.4 ± 0.1	-8.4 ± 0.1	-6.0 ± 0.4	-6.0 ± 0.4	-6.0 ± 0.4	-2.4 ± 0.4	-2.4 ± 0.4	-2.4 ± 0.4	1.1 ± 0.1

When the binding parameters for a given interaction are equal for the three sites, ligand binding showed no cooperativity (i.e., independent binding); when the binding parameters are different, ligand binding showed cooperativity. In all cases, the binding parameters represent site-specific microscopic parameters for each binding site (i.e., intrinsic site-specific binding parameters modulated by cooperativity factors if applicable).

[a] Fraction of active (ligand binding-competent) protein. Parentheses indicate the parameter n was kept fixed during the fitting analysis due to the low binding affinity.

[b] Number of binding sites per protein trimer according to the ligand binding model.

[c] Protein prebound to PALA in the calorimetric cell.

Supplementary Table 3. Primers used in this study.

Purpose	Sequence (5' – 3')
**Cloning of pET28a and pBSK**	
atATC::pET28a NdeI/ XhoI-Forward	cccctcatatgtttgaacttagtgatgtg
atATC::pET28a NdeI/ XhoI-reverse	tcaccaaccgacaagca
**Mutagenesis**	
atATC::pET28a- F161A- Forward	ctgagaacgctagagaggcttcgtctgccgcgaaag
atATC::pET28a- F161A- reverse	ctttcgcggcagacgaagcctctctagcgttctcag
**Construction of ATC amiRNA lines**	
1-I miR-s.	gataatgacaggtatatcggcagtctctcttttgtattcc
1-II miR-a.	gactgccgatatacctgtcattatcaaagagaatcaatga
1-III miR*s.	gactaccgatataccagtcattttcacaggtcgtgatatg
1-IV miR*a	gaaaatgactggtatatcggtagtctacatatatattcc
**Construction of ATC overexpressor lines**	
atATC_VL_fwd	ggggacaagtttgtacaaaaaagcaggcttcaccatgtctattgcatcat
atATC_LV_rev	ggggaccactttgtacaagaaagctgggtctcaccaaccgacaagcagta
BAR for (Basta screening)	aagtccagctgccagaaacc
BAR rev (Basta screening)	accactacatccagacaagc
**Primer for qRT PCR**	
Actin_fwd	cttgcaccaagcagcatgaa
Actin_rev	ccgatccagacactgtacttcctt
ATCase_fwd_RT	tggacgtctataccatcc
ATCase_rev_RT	gcatcactcctaacagat

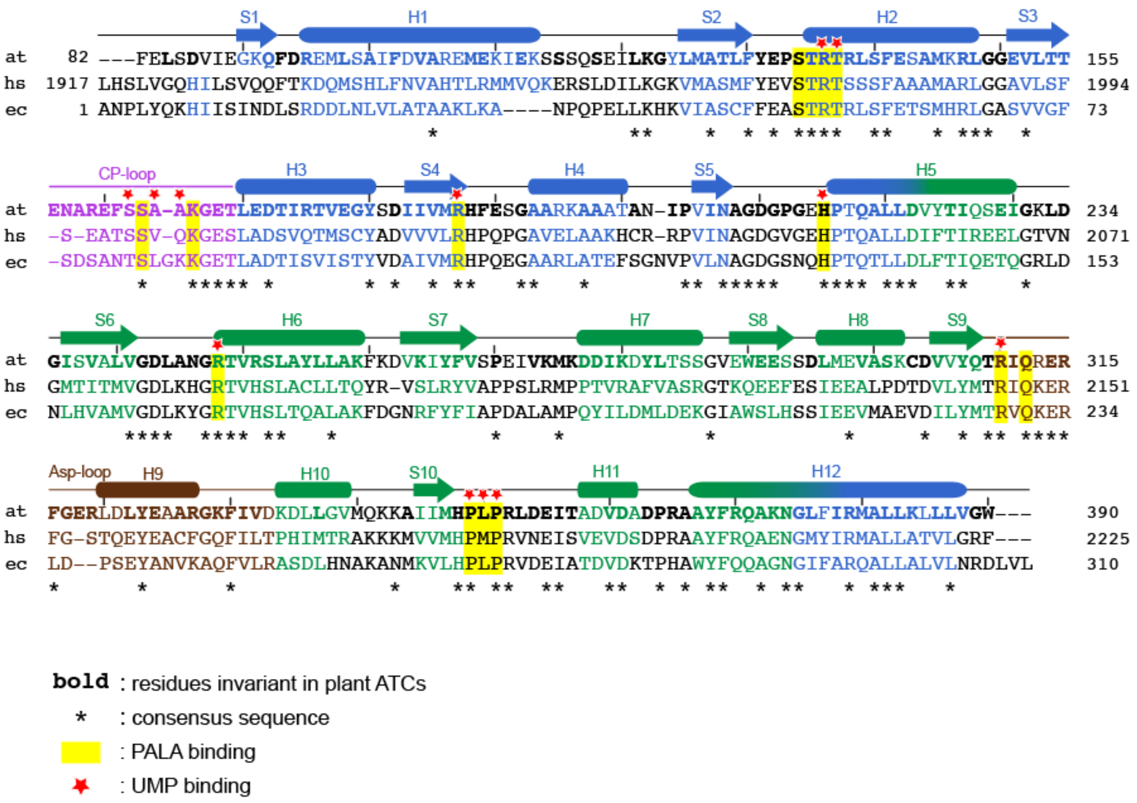

Supplementary Figure 1. ATC sequence alignment. Structure-guided alignment of Arabidopsis (at), human (hs) and *E. coli* (ec) ATC sequences. The chloroplast transit peptide is not included. Asterisks indicate the consensus sequence. Sequence identity between Arabidopsis ATC and human or *E. coli* ATCs is 46% (138 of 302 aa, 97% covery) and 43% (131 of 302 aa, 97% covery), respectively. Conserved active site residues are shown on yellow background and residues interacting with UMP in atATC are indicated with a red star. Residues are colored according to the secondary structure, which is depicted above. α-Helices are shown as cylinders and β-strands as arrows. Residues in bold are found invariant in the following plant ATC sequences: *Pisum sativum* (Q43086 and Q43087), *Phaseolus vulgaris* (Phvul.008G270600.1), *Trifolium pratense* (UPI000844B1D2 and UPI0008432BF2), *Glycine max* (Glyma.02g293500 Glyma.14G021000.1 and Glyma.05G131400.1), *Oryza sativa* (Q9LD61), *Triticum aestivum* (partial sequence aa 122-390, TraesCS5A02G120200.1; EnsemblPlants), *Camellia sinensis* (XP_028078246.1), *Eutrema salsungineum* (XP_006406396.1), *Capsella rubella* (XP_023641307.1), *Raphanus sativus* (XP_018490780.1), *Mircothlaspi erraticum* (A0A6D2KMN0), *Brassica oleracea* (XP_013638479.1), *Brassica napus* (XP_013728634.1), *Pistacia vera* (XP_031265500.1), *Citrus clementina* (XP_006427067.1), *Cajanus cajan* (XP_020237797.1), *Carica papaya* (XP_021906135.1), *Punica granatum* (XP_031407045.1), *Prunus dulcis* (BBH02036.1), *Hevea brasiliensis* (XP_021642880.1), *Vitis vinifera* (RVW30007.1), *Jatropha curcas* (XP_012070892.1), *Manihot esculenta* (XP_021593647.1), *Ricinus communis* (XP_002529466.2), *Prunus persica* (ONI13427.1), *Aquilegia coerulea* (PIA59089.1), *Striga asiatica* (GER28020.1), *Trema orientale* (PON91905.1), *Carpinus fangiana* (KAE8125493.1), *Cucurbita moschata* (XP_022932282.1) and *Aquilegia coerulea* (PIA59089.1).

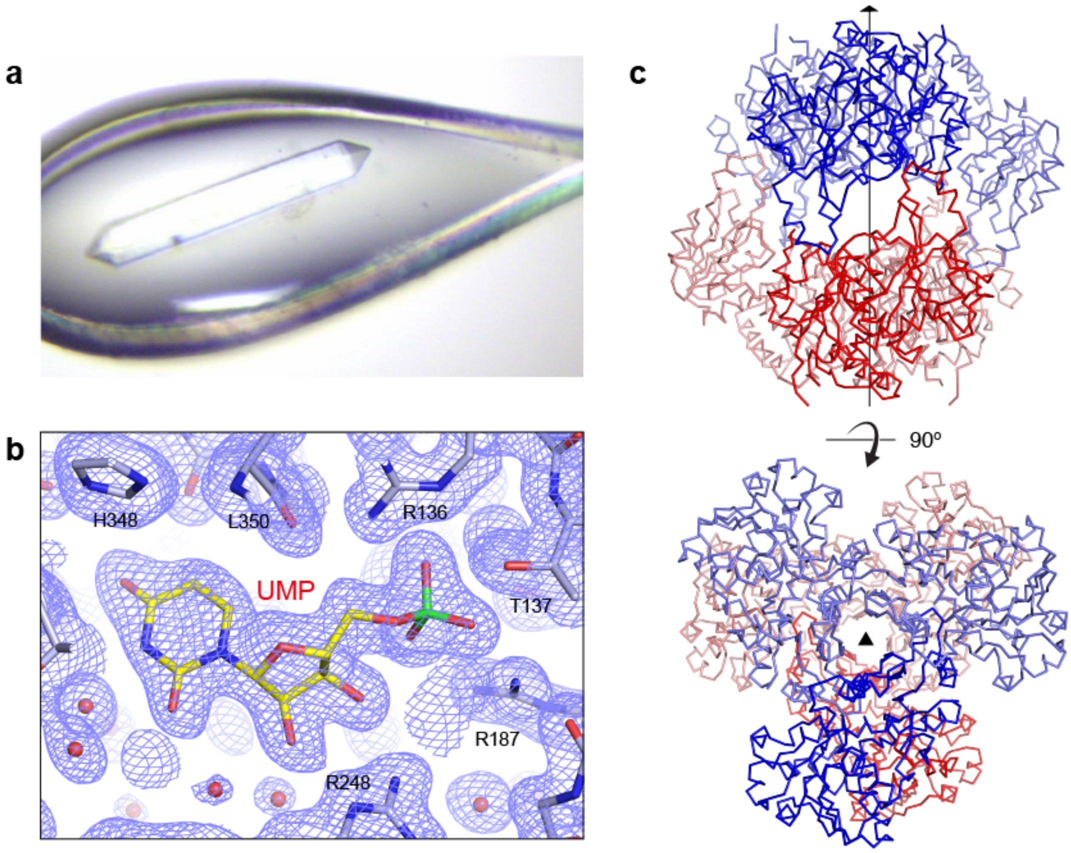

Supplementary Figure 2. Structural determination of Arabidopsis ATC. a atATC crystal mounted on a cryo-loop during diffraction collection. **b** Detail of the active site with UMP bound and 2F_{obs}-F_{calc} electron density map represented in blue mesh. **c** Perpendicular views of the two atATC subunits in the asymmetric unit colored in dark blue and red. Each subunit belongs to a different trimer that forms around the crystallographic three-fold axis. The other subunits in the trimers are depicted in lighter color.

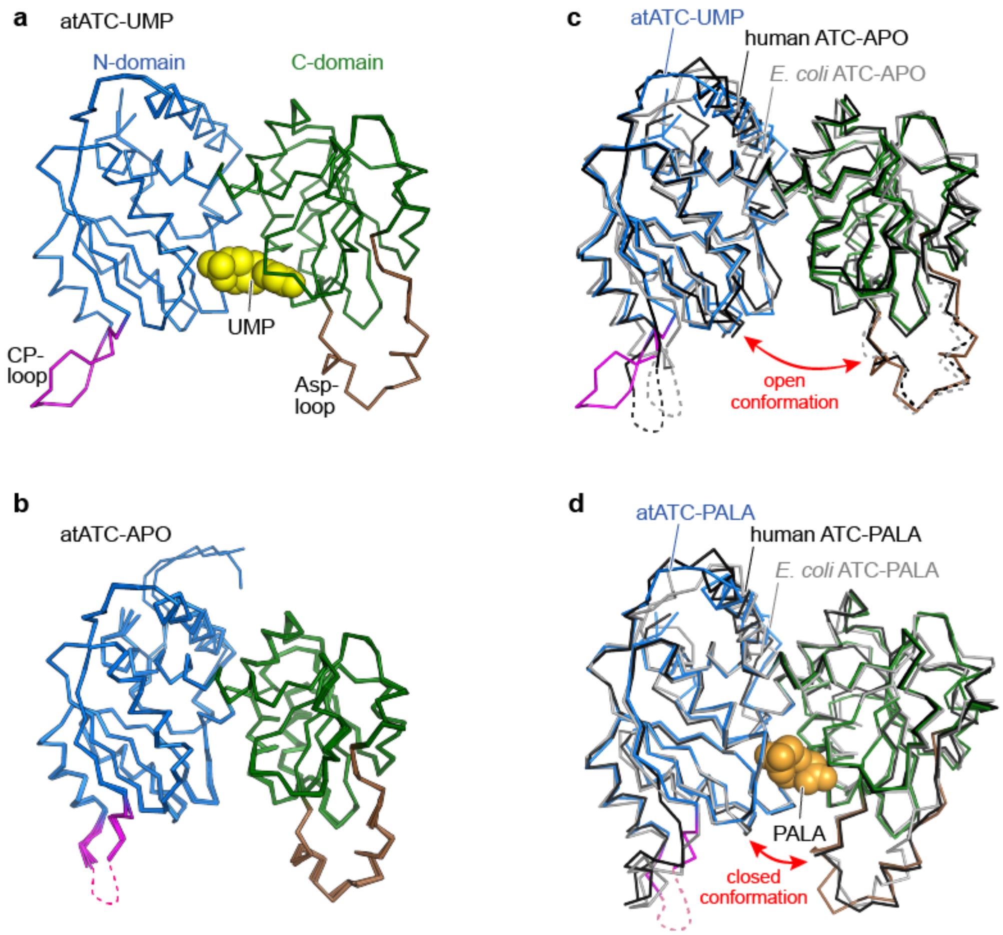

Supplementary Figure 3. Comparison of atATC subunits within the different crystal structures. a Superposition of the two protein subunits in the asymmetric unit of the atATC-UMP crystal. The entire polypeptide chains (311 Cα's) superimpose with a rmsd= 0.24 Å. The subunits are represented in cartoon with the N- and C-domains depicted in blue and green, respectively. The CP-loop and the Asp-loop are colored pink and brown, respectively. UMP is shown as yellow spheres. **b** Similar representation for the superposition of the six subunits in the atATC-APO crystal (rmsd=0.34-0.48 Å for 301-304 Cα's). Dashed line indicates the flexibly disordered CP-loop. **c** Superposition of atATC-UMP subunit with the apo structures of human and *E. coli* ATCs (PDB 5G1O and 3CSU). **d** Superposition of atATC-PALA subunit with the PALA-bound subunits of human and *E. coli* ATCs (PDB 5G1N and 1D09).

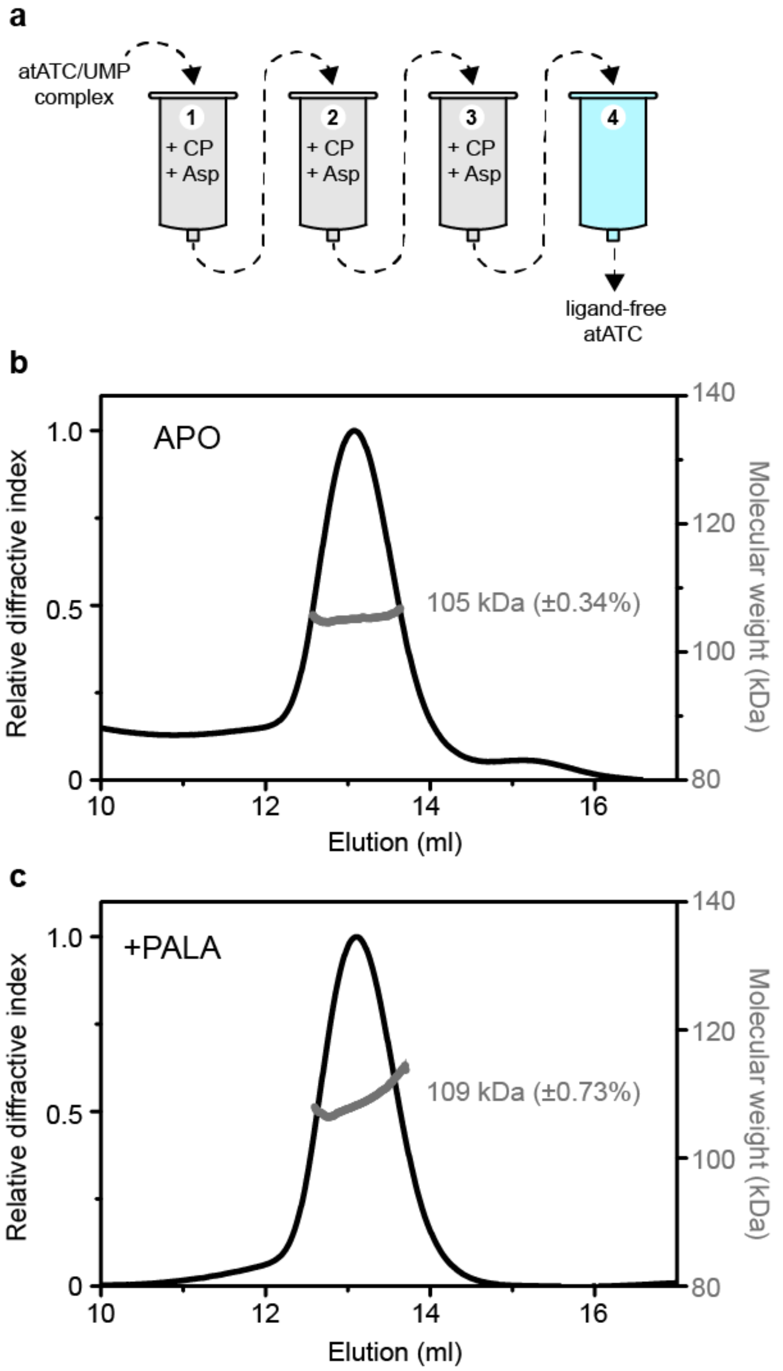

Supplementary Figure 4. atATC free or bound to PALA remains as a homotrimer in solution. a Scheme of the gel filtration procedure to remove UMP from atATC. The sample (2.5 ml at a concentration of 1 mg ml^{-1}) is filtered through three consecutive 5 ml gel-filtration columns (PD-10 GE) equilibrated in a buffer solution containing 5 mM CP and 10 mM Asp. The sample elutes in 3.5 ml and is concentrated in an ultracentrifugation device to 2.5 ml before applying to the next column. In the last step, the sample is applied to a column equilibrated without substrates. The procedure lasts ~1 h and is performed at room temperature. **b,c** SEC-MALS analysis of the UMP-freed sample (**b**) and pre-incubated with PALA (**c**).

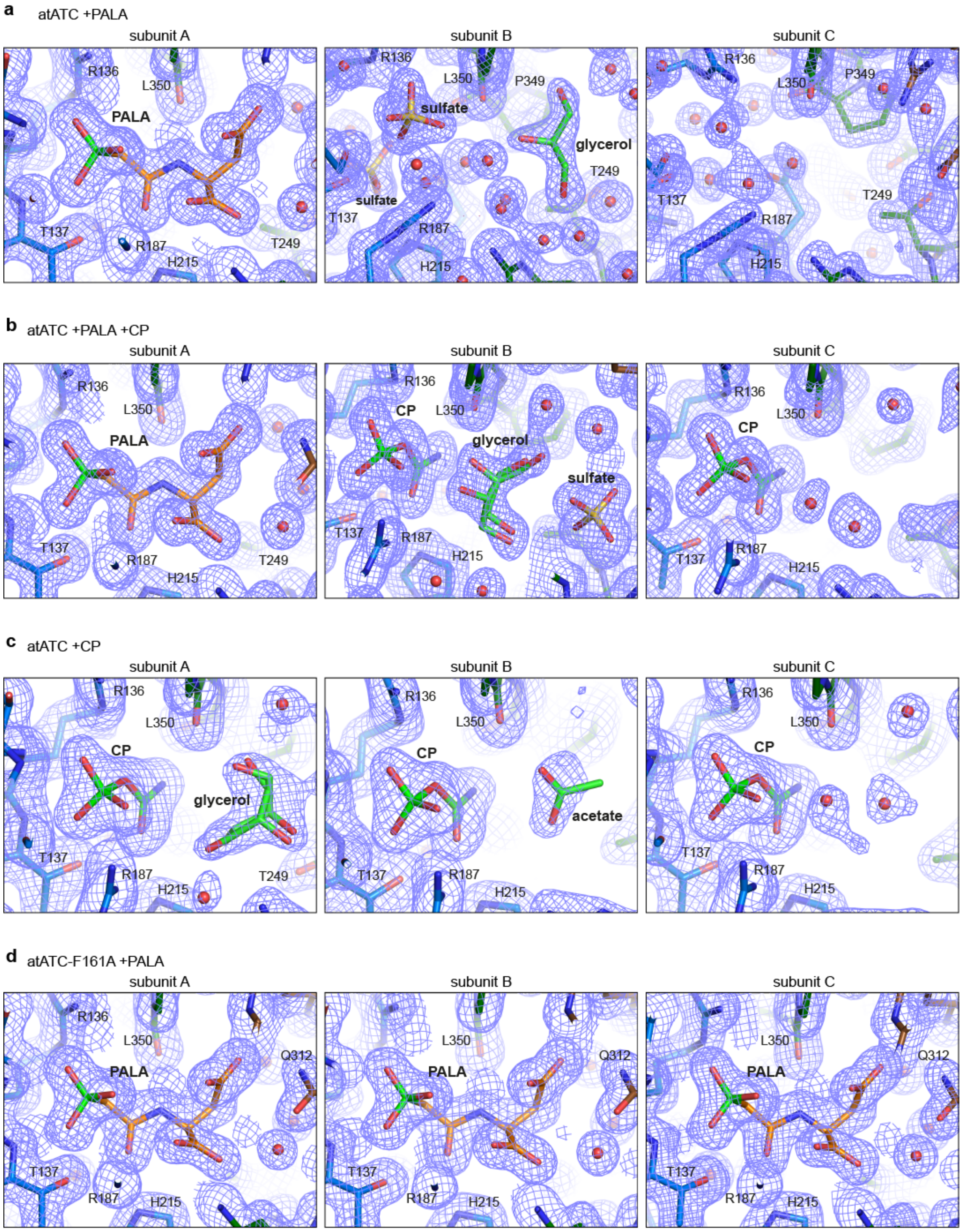

Supplementary Figure 5. atATC active site contents. Detail of the active sites in the atATC-WT trimer crystallized with PALA **(a)**, PALA and CP **(b)** and CP alone **(c)**, and of the atATC-F161A trimer with PALA **(d)**. The $2F_{obs}$-F_{calc} electron density map is represented in blue mesh. Water molecules are shown as red spheres. Glycerol molecules in **b** and **c** were modelled in two alternate conformations to best explain the electron density.

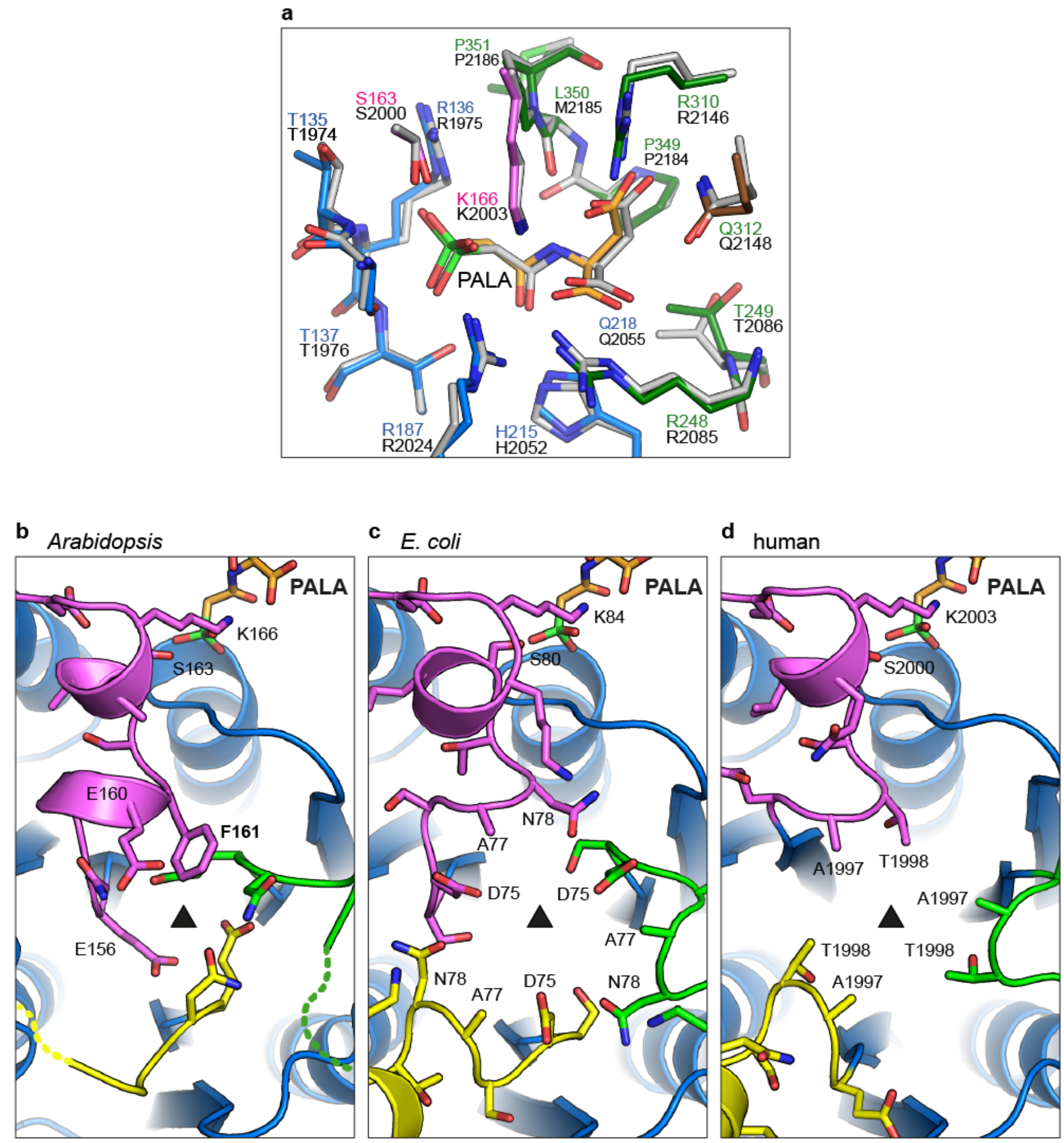

Supplementary Figure 6. PALA binds to only one subunit per trimer. a Superimposition of PALA-bound active sites of atATC and human ATC. atATC residues from the N-domain, the CP-loop or the C-domain are colored with C atoms in blue, pink or green, respectively, whereas PALA is depicted with C atoms in orange. Residue numbers are colored accordingly. The C atoms of human ATC are depicted light grey and residues are labeled in black. **b–d** View along the threefold axis of Arabidopsis (**b**), *E. coli* (**c**) and human (**d**) ATCs bound to PALA. The CP-loops from the three subunits in the trimer are shown in different colors.

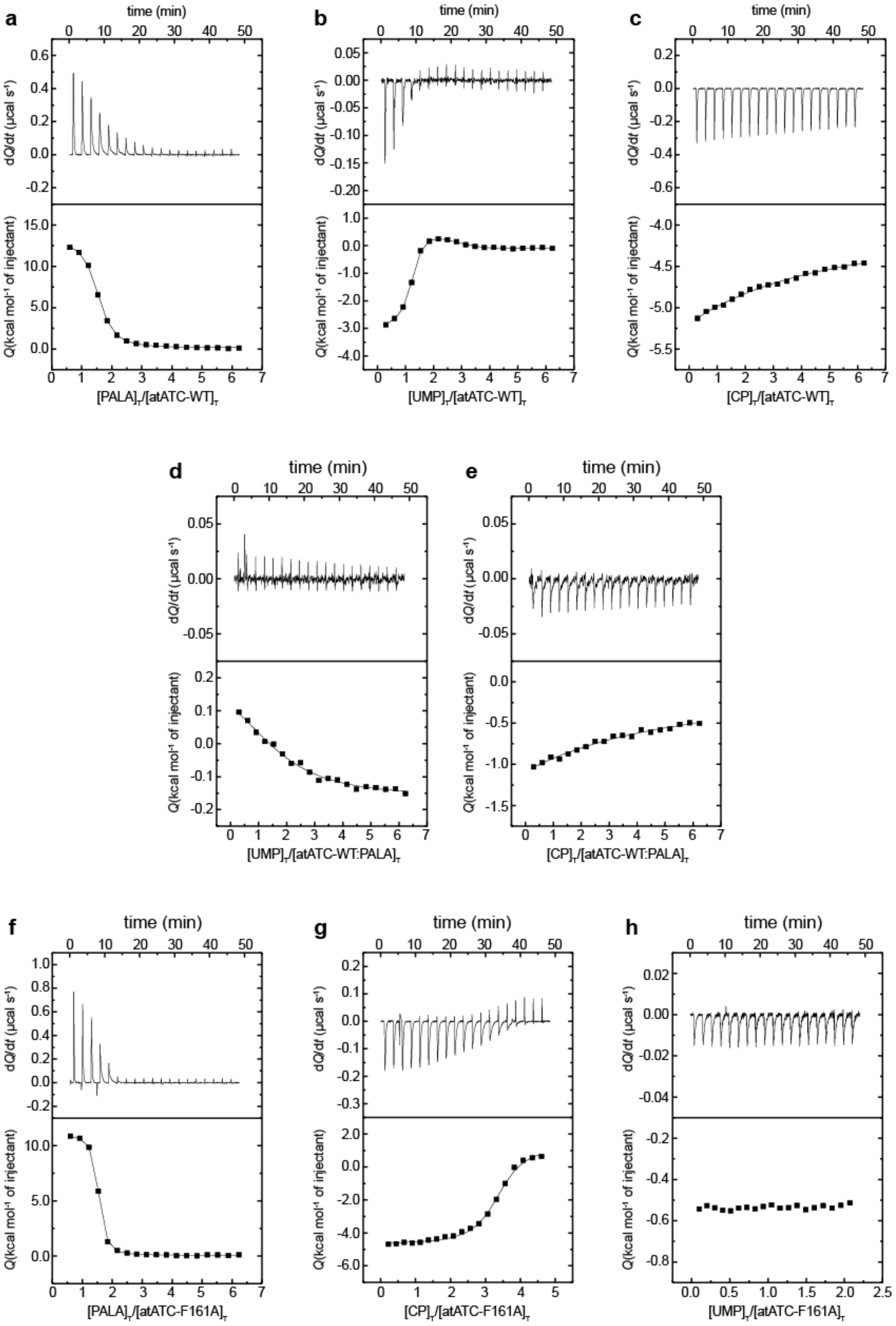

Supplementary Figure 7. ITC data. Calorimetric titrations for the interaction of atATC-WT and atATC-F161A with PALA, UMP and CP. Upper plots show the thermogram (thermal power as a function of time) and lower plots show the binding isotherm (normalized heat per injection as a function of the molar ratio ligand/protein). Non-linear fitting analysis was performed as described in Methods. The CP/atATC-WT titration is characterized by a *c*=0.17, the UMP/atATC-WT:PALA titration by *c*=12, and the CP/atATC-WT:PALA by *c*=0.1, where $c=[\text{Protein}]_T/K_D$. Due to the low affinity for CP/atATC-WT and CP/atATC-WT:PALA interactions, raising the concentration of the ligand would not result in a practical improvement in the shape of the titration, and problems with the dilution of highly concentrated ligands in the syringe would arise. For significantly improving *c*, the cell concentration should be raised to impractical values, and the same factor or larger for the syringe concentration. At low *c*, it is possible to reliably estimate the binding affinity even if a considerable uncertainty affects the enthalpy and the stoichiometry (*Tellinghuisen J. Isothermal titration calorimetry at very low c. Anal Biochem. 2008, 373(2):395-7*).

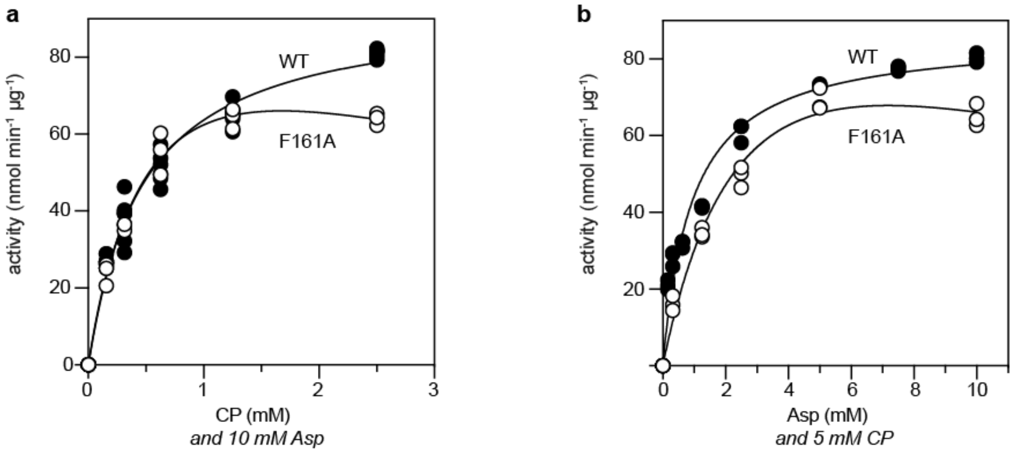

	WT	F161A
**Best-fit values**		
V_{max}	93.23	115.0
$K_{0.5}^{CP}$	0.46	0.62
K_i	>>>	4.52
**Std. error**		
V_{max}	2.53	14.62
$K_{0.5}^{CP}$	0.04	0.13
K_i	-	1.64
**95% confidence interval**		
V_{max}	88.07 - 98.38	83.83 - 146.2
$K_{0.5}^{CP}$	0.39 - 0.54	0.35 - 0.89
K_i	-	1.03 - 8.01
k_{cat} (min^{-1})	3,463	4,273
$k_{cat}/K_{0.5}^{CP}$ (min^{-1} mM^{-1})	7,530	6,891

	WT	F161A
**Best-fit values**		
V_{max}	85.96	128.4
$K_{0.5}^{Asp}$	0.94	3.20
K_i	>>>	16.08
**Std. error**		
V_{max}	2.51	26.71
$K_{0.5}^{Asp}$	0.10	1.03
K_i	-	8.51
**95% confidence interval**		
V_{max}	80.80 - 91.13	71.52 - 185.4
$K_{0.5}^{Asp}$	0.73 - 1.16	1.00 - 5.40
K_i	-	0 - 34.23
k_{cat} (min^{-1})	3,194	4,771
$k_{cat}/K_{0.5}^{Asp}$ (min^{-1} mM^{-1})	3,398	1,491

Supplementary Figure 8. Kinetics of atATC-WT and atATC-F161A. a Dependence of the initial rate (v) of atATC WT and F161A on the concentration of CP **(a)** or Asp **(b)** at fix concentration of the other substrate. The curves were fitted to a Michaelis-Menten equation with an additional term for inhibition by excess substrate: $v = V_{max} \cdot X/K_{0.5} + X(1+X/K_i)$; where X is the substrate concentration, $K_{0.5}$ is the substrate concentration at which v is one-half of the maximum velocity (Vmax) and K_i is the inhibition constant. Best-fitting values are indicated below the graphs. atATC WT does not show inhibition by substrate since the K_i is exceedingly large and thus, obeys the Michaelis-Menten equation and $K_{0.5} = K_M$.

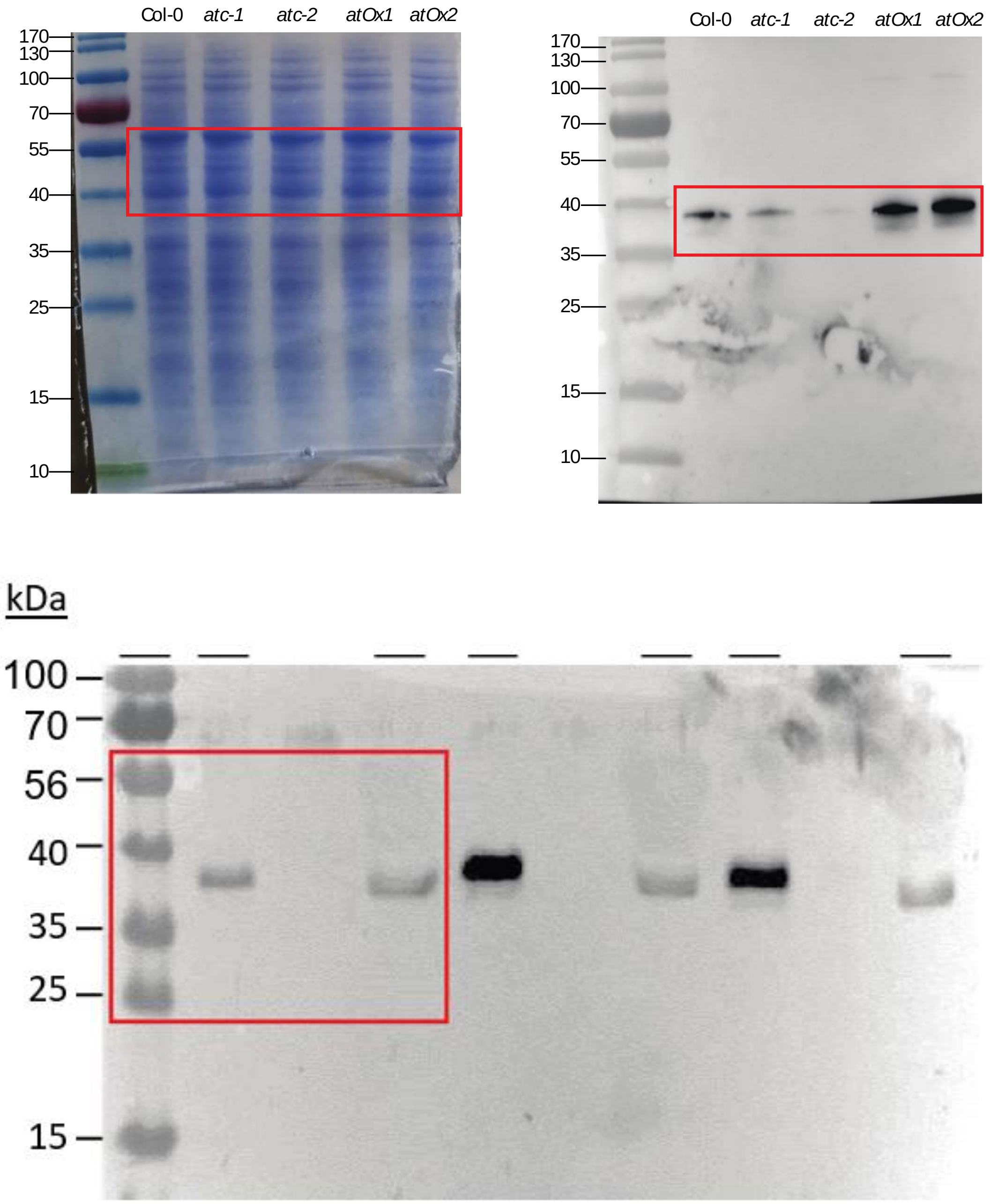

Supplementary Figure 9. Full blot and gels included in main Figure 1 and Figure 2.
