# Supplementary material for: Mechanisms of feedback inhibition and sequential firing of active sites in plant aspartate transcarbamoylase
Source: Nat Commun. 2021 Feb 11;12:947. doi: 10.1038/s41467-021-21165-9 (PMC7878868; doi:10.1038/s41467-021-21165-9)
Supplement: Supplementary file 3 — Reporting Summary [file 41467_2021_21165_MOESM3_ESM.pdf]

nature research

Corresponding author(s): Santiago Ramon-Maiques

Last updated by author(s): Dec 21, 2020

Reporting Summary

Nature Research wishes to improve the reproducibility of the work that we publish. This form provides structure for consistency and transparency in reporting. For further information on Nature Research policies, see our Editorial Policies and the Editorial Policy Checklist.

Statistics

For all statistical analyses, confirm that the following items are present in the figure legend, table legend, main text, or Methods section.

n/a	Confirmed	
☐	☒	The exact sample size (n) for each experimental group/condition, given as a discrete number and unit of measurement
☐	☒	A statement on whether measurements were taken from distinct samples or whether the same sample was measured repeatedly
☐	☒	The statistical test(s) used AND whether they are one- or two-sided<br>*Only common tests should be described solely by name; describe more complex techniques in the Methods section.*
☒	☐	A description of all covariates tested
☒	☐	A description of any assumptions or corrections, such as tests of normality and adjustment for multiple comparisons
☐	☒	A full description of the statistical parameters including central tendency (e.g. means) or other basic estimates (e.g. regression coefficient) AND variation (e.g. standard deviation) or associated estimates of uncertainty (e.g. confidence intervals)
☐	☒	For null hypothesis testing, the test statistic (e.g. F, t, r) with confidence intervals, effect sizes, degrees of freedom and P value noted<br>*Give P values as exact values whenever suitable.*
☒	☐	For Bayesian analysis, information on the choice of priors and Markov chain Monte Carlo settings
☒	☐	For hierarchical and complex designs, identification of the appropriate level for tests and full reporting of outcomes
☒	☐	Estimates of effect sizes (e.g. Cohen's d, Pearson's r), indicating how they were calculated

Our web collection on statistics for biologists contains articles on many of the points above.

Software and code

Policy information about availability of computer code

Data collection	X-ray dataset were collected and processed with XDS and autoPROC
Data analysis	Crystallographic data were analyzed with CCP4 (version 7.1; including PHASER, COOT and REFMAC5) and PHENIX (version 1.8). Data from other experiments were analyzed and plot with GraphPad Prism8 and Origin7. Crystal structures were analyzed and represented with PyMOL version 2.0.

For manuscripts utilizing custom algorithms or software that are central to the research but not yet described in published literature, software must be made available to editors and reviewers. We strongly encourage code deposition in a community repository (e.g. GitHub). See the Nature Research guidelines for submitting code & software for further information.

Data

Policy information about availability of data

All manuscripts must include a data availability statement. This statement should provide the following information, where applicable:

- Accession codes, unique identifiers, or web links for publicly available datasets
- A list of figures that have associated raw data
- A description of any restrictions on data availability

The structural data (coordinates and structure factors) that support the findings of this study are deposited in the Protein Data Bank (PDB) under accession codes 6YPO (+UMP), 6YY1 (APO), 6YS6 (+PALA), 6YSP (+PALA +CP), 6YVB (+ CP), 6YWJ (F161A +UMP) and 6YW9 (F161A +PALA). The ITC data that support the findings of this study are available from the corresponding author upon reasonable request. A separate source data file contains raw data underlying Figure 1c-g, Figure 2b,c, Figure3a, Figure 5a-c, Supplementary Figure 4b,c, and Supplementary Figure 8; it also contains full blots and gels of cropped images shown in Figure 1 and Figure 2 (see Supplementary Figure 9).

Field-specific reporting

Please select the one below that is the best fit for your research. If you are not sure, read the appropriate sections before making your selection.

☒ Life sciences ☐ Behavioural & social sciences ☐ Ecological, evolutionary & environmental sciences

For a reference copy of the document with all sections, see nature.com/documents/nr-reporting-summary-flat.pdf

Life sciences study design

All studies must disclose on these points even when the disclosure is negative.

Sample size	Individual plants (mutants and controls) were grown side by side, at least three per line, but usually more than 8. When working with plants, it is intended to be as close to natural growth conditions as possible. Therefore, we grow plants in individual pots in soil. Only if treatments require other growth techniques (hydroponics, Agar plates etc.) these are used. Sample size of soil grown plants is always limited by growth room capacity and the time needed for harvest. In one experiment the minimum number of samples per genotype is three plants (=biological replicates.). For 5 genotypes 15 plants are grown simultaneously. Whole experiments are replicated three times (= 9 biol. replicates in total). It can happen that samples are lost during the experiment, explaining sample size differences between genotypes. We regard this sample size as sufficient as long as the statistical analysis applied supports significance of the analysis. These considerations are based on "Poorter et al., 2012 "The art of growing plants for experimental purposes: a practical guide for the plant biologist". Functional Plant Biology, 39, 821-838.
Data exclusions	No data were excluded
Replication	All experiments were replicated at least three times consecutively. This replication serves to identify and balance unintended variations in growth rooms (light, temperature, watering).
Randomization	Allocation was random
Blinding	Blinding was not possible because of apparent mutant phenotypes

Reporting for specific materials, systems and methods

We require information from authors about some types of materials, experimental systems and methods used in many studies. Here, indicate whether each material, system or method listed is relevant to your study. If you are not sure if a list item applies to your research, read the appropriate section before selecting a response.

Materials & experimental systems

n/a	Involved in the study	
☐	☒	Antibodies
☒	☐	Eukaryotic cell lines
☒	☐	Palaeontology and archaeology
☐	☒	Animals and other organisms
☒	☐	Human research participants
☒	☐	Clinical data
☒	☐	Dual use research of concern

Methods

n/a	Involved in the study	
☒	☐	ChIP-seq
☒	☐	Flow cytometry
☒	☐	MRI-based neuroimaging

Antibodies

Antibodies used	Rabbit polyclonal antiserum raised against recombinant ATC protein was produced and purified in the laboratory of Torsten Möhlmann (custom made antibody) and used at a 1:5.000 dilution. As secondary antibody, the (anti rabbit) HRP linked (Promega #W4011, Walldorf, Germany) was used at a 1:10.000 dilution.
Validation	The pre-immune serum was tested against recombinant protein and in plant extracts without any observed cross-reactivity

Animals and other organisms

Policy information about studies involving animals; ARRIVE guidelines recommended for reporting animal research

Laboratory animals	The study did not involve laboratory animals
Wild animals	The study did not involve wild animals
Field-collected samples	The study did not involved field-collected samples

Ethics oversight	No ethical approval or guidance was needed since our study involved the work with Arabidopsis plants.

Note that full information on the approval of the study protocol must also be provided in the manuscript.
